# Timing of Dexamethasone Initiation and Its Impact on the Outcome of COVID-19 Patients

**DOI:** 10.7759/cureus.72983

**Published:** 2024-11-04

**Authors:** Jamila Alonazi, Najla Alrasheed, Saad Aljabr, Khalaf Albaqami, Khalid Alshallal, Saif A Alsemairi, Fahad AlBaqami, Nawaf F Alnufaie, Faisal A Bin Talib

**Affiliations:** 1 Department of Internal Medicine, King Abdulaziz Medical City, Riyadh, SAU; 2 Division of Internal Medicine, Department of Medicine, King Abdulaziz Medical City, Ministry of National Guard Health Affairs (MNGHA), Riyadh, SAU; 3 Division of Medicine, Department of Medicine, King Abdulaziz Medical City, Ministry of National Guard Health Affairs (MNGHA), Riyadh, SAU; 4 Department of Radiology, King Faisal Specialist Hospital and Research Centre, Riyadh, SAU

**Keywords:** corticosteroid, covid-19, dexamethasone, dexamethasone initiation time, icu

## Abstract

Introduction

COVID-19 emerged in Wuhan in December 2019 and was declared a pandemic in March 2020. Severe cases manifest with respiratory distress. Corticosteroids, initially debated, are now recommended for severe cases following the RECOVERY (Randomised Evaluation of COVID-19 Therapy) trial findings. The timing of administration impacts outcomes, with earlier use potentially improving mortality and ICU stays. Regional studies on timing in severe cases are lacking, warranting further investigation.

Methodology

This retrospective cohort study was conducted at the Medical Department of King Abdulaziz Medical City (KAMC), Riyadh, Saudi Arabia. Data were extracted from the BestCare database using a customized data collection sheet. Data were cleaned in Excel (Microsoft Corporation, Redmond, WA) and analyzed in IBM SPSS (IBM Corp., Armonk, NY).

Results

Our study included 791 COVID-19 patients with 43.1% being female (n = 341) and 56.9% being male (n = 450). The mean age was 69.5 years (SD = 16.1). Regarding BMI, 52.4% (n = 414) were obese. Most admissions were from the emergency department (90.6%, n = 717). Dexamethasone was administered to 80.3% (n = 635) of patients, with 53.0% (n = 419) receiving it early. Patients receiving early dexamethasone had significantly higher discharge rates (p < 0.001). Mortality was higher among those receiving late dexamethasone initiation (52.6%, p = 0.256). Moreover, there was an 87.5% death rate for doses >6 mg (p < 0.001). Intravenous administration was associated with higher mortality (62.3%, p < 0.001). Males had a higher likelihood of discharge (OR = 1.426, p = 0.043). Age and ventilation needs were strong mortality predictors (OR = 1.040, p < 0.001 and OR = 17.620, p < 0.001, respectively). Higher BMI slightly reduced mortality risk (OR = 0.978, p = 0.049).

Conclusion

Our study highlights significant associations between dexamethasone timing, dosage, and route of administration with COVID-19 outcomes. Early dexamethasone use correlated with higher discharge rates, while late initiation and higher doses were linked to increased mortality. Age and ventilation needs were critical predictors, with BMI showing a nuanced effect on mortality risk.

## Introduction

Despite millions of deaths globally from coronavirus disease 2019 (COVID-19), few effective treatments are currently available. COVID-19 is a viral infection caused by severe acute respiratory syndrome coronavirus 2 (SARS-CoV-2), a member of the *Betacoronavirus* genus, similar to Middle East respiratory syndrome-related coronavirus (MERS-CoV) and severe acute respiratory syndrome coronavirus (SARS-CoV) [1]. It was first identified in Wuhan, Hubei Province, China in December 2019. In March 2020, the WHO declared COVID-19 a global pandemic, with the disease affecting over 210 countries in the world [1,2]. The first documented case of COVID-19 was reported on March 2, 2020, in Saudi Arabia. By April 22, 2021, the total number of COVID-19 cases in Saudi Arabia had reached 408,038, with 6,858 deaths, resulting in a mortality rate of 1.7% [3]. Most cases of the infection cause only mild symptoms, but a substantial number of patients still experience severe respiratory distress and hypoxemia, and require ventilatory support [1,4].

The hallmark pathophysiological characteristics of severe COVID-19 include acute pneumonia with bilateral multifocal alveolar opacities, sometimes accompanied by pleural effusion [5]. Autopsy findings in such patients have revealed diffuse alveolar damage, inflammatory infiltrates, and microvascular thrombosis [6]. Corticosteroids are generally used as immunosuppressants; however, they are also widely used for the treatment of inflammatory conditions [7]. Currently, they are extensively used in syndromes related to COVID-19, such as MERS-CoV, severe influenza, and community-acquired pneumonia [8,9]. However, during the early days of the pandemic, the role of corticosteroids in the treatment of COVID-19 patients was unclear and the WHO recommended against their use, citing their severe side effects and potential impact on prolonging viral shedding [1]. With the emergence of new evidence on the efficacy of corticosteroids in COVID-19, the WHO recommendations changed. The current practice strongly recommends the use of systemic corticosteroids in severe and critical patients [1].

The strongest evidence in this regard was provided by the RECOVERY (Randomised Evaluation of COVID-19 Therapy) trial that included 2,104 patients who received dexamethasone and 4,321 patients who received usual care. The findings of the trial revealed that 482 patients (22.9%) in the dexamethasone group and 1,110 patients (25.7%) in the usual care group died within 28 days after randomization. The incidence of death was lower in the dexamethasone group compared to the usual care group among patients receiving invasive mechanical ventilation (29.3% vs. 41.4%) and among those receiving oxygen without invasive mechanical ventilation (23.3% vs. 26.2%). However, there was no reduction in mortality among those who were not receiving respiratory support at randomization (17.8% vs. 14.0%) [4]. The outcome of corticosteroid use in COVID-19 is probably associated with the timing of their administration. Current evidence from the RECOVERY trial suggests that the greater mortality benefit of dexamethasone is seen in patients who are receiving respiratory support and among those who were recruited > seven days after the onset of their symptoms [4].

This evidence supports the current hypothesis that the natural history of COVID-19 includes an initial stage of viral replication followed by a second stage of immunopathological hyperinflammatory response [5]. Furthermore, recent literature suggests that earlier use of dexamethasone in critically ill patients was associated with lower mortality rates and shorter intensive care unit length of stay [10-12]. Despite these findings, there are currently no clear recommendations on when corticosteroids should be initiated in severe COVID-19 patients. Therefore, the present study aims to evaluate the impact of the initial timing of corticosteroid therapy on morbidity, mortality, and hospital stay in severe COVID-19 patients.

## Materials and methods

Study design and setting

This is a retrospective cohort study of all patients diagnosed with COVID-19 at King Abdulaziz Medical City (KAMC), Riyadh, Saudi Arabia. Data were collected from all the patients admitted for COVID-19 from August 1, 2020, to August 29, 2021.

Study subjects

The inclusion criteria for the study comprised all consecutive severe COVID-19 patients admitted to KAMC, who developed hypoxemia. Only patients above 18 years of age were included. All eligible patients had confirmed SARS-CoV-2 infection from a respiratory tract sample using a reverse transcription-polymerase chain reaction (RT-PCR) assay. The exclusion criteria included patients who had non-confirmed SARS-CoV-2 infection by RT-PCR assay. Furthermore, patients with no data at baseline and those who had do-not-resuscitate (DNR) orders were excluded. Patients were excluded if they did not meet the outcomes of either death or ICU discharge by the end of the data collection period, or if they had recently received systemic corticosteroid treatment before contracting COVID-19.

Sample size and sampling

The study included all patients who met the inclusion criteria and were treated with dexamethasone as part of their management plan during the study period, estimated at approximately 300-400 patients. Patients were identified from the electronic health records at KAMC.

Data collection

Data were collected through a retrospective chart review of electronic medical records from BestCare. Demographic data for the study population was collected, including age, sex, body mass index (BMI), smoking status, and underlying diseases (hypertension, diabetes, cerebrovascular disease, cardiovascular disease, chronic lung disease, chronic kidney disease, chronic liver disease, and cancer). Individuals with a BMI of less than 18.5 are considered underweight. A BMI ranging from 18.5 to 24.9 is categorized as normal weight, while those with a BMI of 25.0 to 29.9 are classified as overweight. Obesity is further divided into three classes: class I includes a BMI of 30.0 to 34.9, class II ranges from 35.0 to 39.9, and class III, also known as severe or morbid obesity, is defined by a BMI of 40.0 or higher. In addition, data regarding the presence of pneumonia, and complications such as length of stay and in-hospital mortality were collected. Treatment information included the time of initiation of dexamethasone, the total dose of dexamethasone, the duration of dexamethasone treatment, and the time interval from symptom onset or diagnosis to initiation of dexamethasone administration. Patients were classified as having severe COVID-19 if they required mechanical ventilation, supplemental oxygen, or ICU admission during their hospital stay. The timing of dexamethasone administration was categorized as early if initiated within the first 24 hours of admission and as late if started after the initial 24-hour period.

Data management and analysis plan

All data were obtained directly from BestCare and were entered into Microsoft Excel (Microsoft Corporation, Redmond, WA). The dataset was subsequently imported into SPSS version 22 (IBM Corp., Armonk, NY) for statistical analysis. Numerical variables were described as mean and standard deviation, whereas the categorical variables were described as frequencies and proportions. For categorical variables, chi-square or Fisher's exact test was used, as appropriate, to evaluate relationships between groups. The Mann-Whitney U test was used to assess the difference between two continuous variables. A p-value < 0.05 was considered statistically significant.

Ethical considerations

Due to the retrospective nature of the study, there was no direct contact between investigators and patients. Therefore, it was not possible to obtain informed consent from each participant. However, patient confidentiality and data privacy were strictly maintained by anonymizing patient data. Personal details such as names, medical records, and other identifiers were replaced with numbers. Access to the data was limited to the investigators only.

## Results

**Figure 1:**
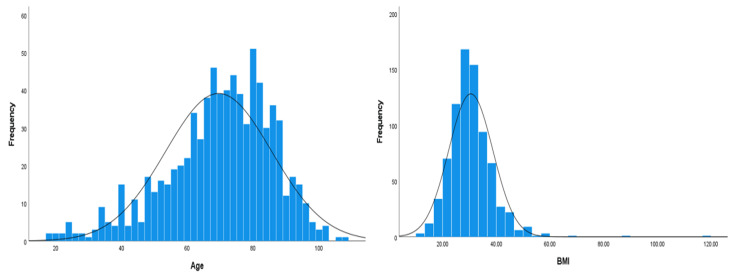
Normality distribution curves for age and BMI.

Our study included 791 COVID-19 patients, out of which 43.1% (n = 341) were female, while 56.9% (n = 450) were male. Age distribution showed that 34.1% (n = 270) were younger than 65 years, 39.6% (n = 313) were between 65 and 80 years, and 26.3% (n = 208) were older than 80 years, with a mean age of 69.5 years (SD = 16.1). The age of the patients ranged from 18 to 108 years (the normality plot of age is shown in Figure 1). Regarding BMI, 3.8% (n = 30) were underweight, 18.1% (n = 143) had normal weight, 25.8% (n = 204) were overweight, and 52.4% (n = 414) were classified as obese (class 1: 29.5%, n = 233; class 2: 13.9%, n = 110; class 3: 9%, n = 71), with a mean BMI of 30.5 (SD = 8.2). The BMI ranged from 10.3 to 117.2 (the normality plot of BMI is shown in Figure 1).

Most admissions were from the emergency department, accounting for 90.6% (n = 717), while 9.2% (n = 73) were admitted from other departments (Table 1).

**Table 1:** Sociodemographic and other surgical parameters of COVID-19 patients.

		Frequency (n = 791)	Percent
Gender	Female	341	43.1
	Male	450	56.9
Age	<65 years	270	34.1
	65-80 years	313	39.6
	>80 years	208	26.3
	Mean (SD)	69.5 (16.1)	
	Range	18-108	
BMI	Underweight	30	3.8
	Normal	143	18.1
	Overweight	204	25.8
	Obese class 1	233	29.5
	Obese class 2	110	13.9
	Obese class 3	71	9.0
	Mean (SD)	30.5 (8.2)	
	Range	10.3-117.2	
Admission of COVID-19 cases from	Emergency	717	90.6
	Other department	73	9.2

Table 2 shows the characteristics of dexamethasone usage and outcomes in COVID-19 patients. Notably, out of 791 COVID-19 patients, 19.7% (n = 156) did not receive dexamethasone, while 53.0% (n = 419) received it early, and 27.2% (n = 215) received it late. For those given dexamethasone, 75.1% (n = 594) were administered a 6 mg dose, with lesser frequencies for doses under 6 mg (2.9%, n = 23) and over 6 mg (2.1%, n = 17). Dexamethasone was administered for less than 10 years in 35.7% (n = 282) of cases and for more than 10 years in 44.1% (n = 349). Routes of administration included intramuscular (7.6%, n = 60), oral (15.0%, n = 119), and intravenous (57.9%, n = 458). Oxygen was required by 81.2% (n = 642) of patients, and 45.9% (n = 363) required ventilation, with 13.1% (n = 104) using bilevel positive airway pressure (BiPAP) and 0.6% (n = 5) using continuous positive airway pressure (CPAP). Severe cases needing ventilation or oxygen comprised 81.9% (n = 648) of patients. Outcomes included discharge against medical advice (2.5%, n = 20), discharge home (55.6%, n = 440), and deceased (41.8%, n = 331).

**Table 2:** Different characteristics of Dexamethasone usage, other parameters and outcome of COVID-19 patients IM: intramuscular; PO: oral; IV: intravenous; BiPAP: bilevel positive airway pressure; CPAP: continuous positive airway pressure.

		Frequency (n = 791)	Percent
Dexamethasone administration	No	156	19.7
	Yes	419	53.0
If yes, administration time	Early	419	53.0
	Late	215	27.2
Dose of dexamethasone	<6 mg	23	2.9
	6 mg	594	75.1
	>6 mg	17	2.1
Duration of dexamethasone	<10 years	282	35.7
	>10 years	349	44.1
Route of administration	IM	60	7.6
	PO	119	15.0
	IV	458	57.9
O_2_ requirement	No	148	18.7
	Yes	642	81.2
Ventilation requirement	No	428	54.1
	Yes	363	45.9
If yes, type of ventilation	BiPAP	104	13.1
	CPAP	5	.6
Severe COVID-19 cases (require ventilation or O_2_ or both)	No	143	18.1
	Yes	648	81.9
Outcome	Discharge against medical advice	20	2.5
	Discharge home	440	55.6
	Deceased/died	331	41.8

Table 3 shows the association between dexamethasone-related parameters and outcome of COVID-19 patients such as dexamethasone dose, O2 requirement, ventilation requirement, and ventilation type. Gender and admission source did not significantly affect outcomes (p = 0.553 and p = 0.592, respectively). However, dexamethasone initiation showed a significant association (p < 0.001); patients not receiving dexamethasone had a higher discharge rate (84.1%, n = 127) and low mortality rate (15.9%, n = 24) compared to those who received it (50.5%, n = 313). Early initiation of dexamethasone did not significantly affect outcomes from late initiation (p = 0.256). Dexamethasone dose and duration also showed significant associations. Patients receiving <6 mg had a 60.9% discharge rate (p = 0.006), while those on >6 mg had an 87.5% death rate. Administration duration of <10 years resulted in a higher discharge rate (63.0%, p < 0.001) compared to >10 years, which shows a higher death rate (59.4%). Route of administration showed significant differences (p < 0.001), with intramuscular (74.1%) and oral (87.9%) routes having higher discharge rates compared to intravenous (37.7%). Oxygen and ventilation requirements were significant predictors of outcomes (p < 0.001). Patients not requiring oxygen had a 94.4% discharge rate, while those needing ventilation had a 78.2% death rate. Ventilation type (BiPAP vs. CPAP) did not show a significant difference (p = 1.000).

**Table 3:** Association between characteristics of dexamethasone usage and outcome of COVID-19 patients. ^a^ Chi-square test; ^b^ Fisher’s exact test.

			Outcome		P-value
			Discharged home	Death/deceased	
Gender	Female	N	186	147	0.553^a^
		%	55.9%	44.1%	
	Male	N	254	184	
		%	58.0%	42.0%	
Admission source	Emergency	N	401	297	0.592^a^
		%	57.4%	42.6%	
	Other department	N	39	33	
		%	54.2%	45.8%	
Dexamethasone initiation	No	N	127	24	<0.001^a^
		%	84.1%	15.9%	
	Yes	N	313	307	
		%	50.5%	49.5%	
Dexamethasone initiation time	Early	N	213	195	0.256^a^
		%	52.2%	47.8%	
	Late	N	100	111	
		%	47.4%	52.6%	
Dexamethasone dose	<6 mg	N	14	9	0.006^a^
		%	60.9%	39.1%	
	6 mg	N	296	284	
		%	51.0%	49.0%	
	>6 mg	N	2	14	
		%	12.5%	87.5%	
Duration of administration	<10 years	N	174	102	<0.001^a^
		%	63.0%	37.0%	
	>10 years	N	138	202	
		%	40.6%	59.4%	
Route of administration	IM	N	43	15	<0.001^a^
		%	74.1%	25.9%	
	PO	N	102	14	
		%	87.9%	12.1%	
	IV	N	169	279	
		%	37.7%	62.3%	
O_2_ requirement	No	N	135	8	<0.001^a^
		%	94.4%	5.6%	
	Yes	N	305	323	
		%	48.6%	51.4%	
Ventilation requirement	No	N	362	51	<0.001^a^
		%	87.7%	12.3%	
	Yes	N	78	280	
		%	21.8%	78.2%	
Ventilation type	BiPAP	N	50	52	1.000^b^
		%	49.0%	51.0%	
	CPAP	N	2	3	
		%	40.0%	60.0%	

**Figure 2:**
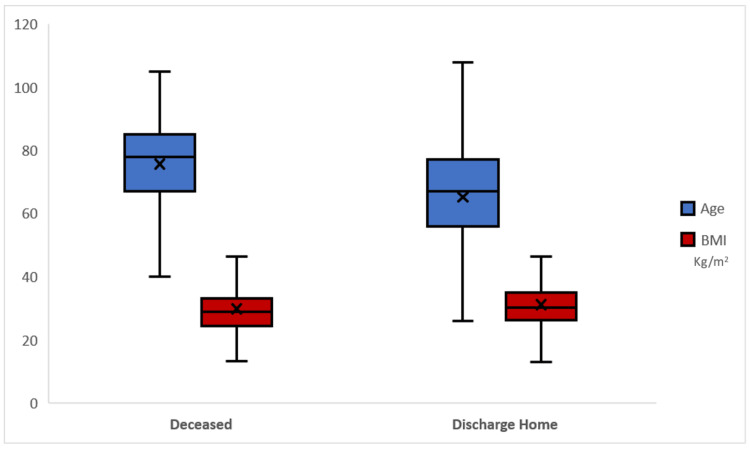
Graphical representation of age and BMI difference according to outcome.

Table 4 and Figure 2 show the age and BMI differences between outcomes in COVID-19 patients. Notably, the mean age of patients discharged home was 65.12 years (SD = 16.17), while the mean age of deceased patients was significantly higher at 75.60 years (SD = 13.93), with a p-value of <0.001, indicating a statistically significant difference. The overall mean age of the 791 patients was 69.51 years (SD = 16.12). Regarding BMI, patients discharged home had a mean BMI of 31.06 (SD = 8.20), compared to a mean BMI of 29.73 (SD = 8.16) for deceased patients. This difference was also statistically significant, with a p-value of 0.025. The overall mean BMI for all patients was 30.50 (SD = 8.20).

**Table 4:** Age and BMI differences between different outcomes. ^a^ Mann-Whitney U test.

		N	Mean	Std. deviation	Sig. value ^a^
Age	Discharge home	460	65.12	16.17	<0.001
	Deceased	331	75.60	13.93	
	Total	791	69.51	16.12	
BMI	Discharge home	460	31.06	8.20	0.025
	Deceased	331	29.73	8.16	
	Total	791	30.50	8.20	

Table 5 shows the result of the binary logistic regression model, which identified several significant predictors of mortality among severe COVID-19 patients. The covariates were determined based on their clinical significance and established associations with mortality in severe COVID-19 patients. Males were found to have a significantly lower risk of mortality compared to females (Exp(B) = 0.658, p = 0.017) due to severe infection after dexamethasone usage. Age was a critical factor, as each additional year increased the odds of mortality significantly (Exp(B) = 1.040, p < 0.001). Higher BMI was associated with a slight reduction in mortality risk (Exp(B) = 0.978, p = 0.049). Late initiation of dexamethasone therapy nearly doubled the risk of mortality (Exp(B) = 1.935, p = 0.005), while intravenous administration of dexamethasone significantly increased the risk (Exp(B) = 2.404, p < 0.001). The need for ventilation was the strongest predictor, drastically increasing the odds of mortality by over 17 times (Exp(B) = 17.620, p < 0.001). Non-significant predictors included dexamethasone dose, duration of dexamethasone use, and oxygen requirement, as their p-values were above the threshold of 0.05.

**Table 5:** Adjusted predictors of mortality among severe COVID-19 patients after dexamethasone usage (binary logistic regression model).

	B	Sig.	Exp(B)	95% CI	
				Lower	Upper
Gender (male)	-0.418	0.017	0.658	0.467	0.928
Age	0.039	0.000	1.040	1.027	1.052
BMI	-0.022	0.049	0.978	0.957	1.000
Dexamethasone initiation (late)	0.660	0.005	1.935	1.219	3.073
Dexamethasone dose	0.496	0.315	1.642	0.624	4.319
Dexamethasone duration	-0.220	0.364	0.802	0.498	1.292
Route of administration (intravenous vs. other)	0.877	0.000	2.404	1.675	3.450
O_2_ requirement	1.455	0.153	4.283	0.581	31.552
Ventilation requirement	2.869	0.000	17.620	10.654	29.140

Table 6 shows the result of the binary logistic regression model, which identified several significant predictors of discharge among severe COVID-19 patients. Male patients had a higher likelihood of being discharged home compared to females, with an odds ratio (Exp(B)) of 1.426 (p = 0.043). Age was inversely related to the likelihood of discharge, with each additional year decreasing the odds of discharge (Exp(B) = 0.962, p < 0.001). Higher BMI slightly increased the odds of discharge, with an odds ratio of 1.022 (p = 0.050). Patients who received dexamethasone late were less likely to be discharged home, with an odds ratio of 0.574 (p = 0.016). The route of administration also played a significant role; those who received dexamethasone intravenously had a lower likelihood of being discharged home (Exp(B) = 0.415, p < 0.001). The need for ventilation was a strong negative predictor of discharge, with the odds of discharge being drastically lower for ventilated patients (Exp(B) = 0.070, p < 0.001). Non-significant predictors included dexamethasone dose and duration, and oxygen requirement, as their p-values were above the threshold of 0.05.

**Table 6:** Adjusted predictors of home discharge among severe COVID-19 patients after dexamethasone usage (binary logistic regression model).

	B	Sig.	Exp(B)	95% CI	
				Lower	Upper
Gender (male)	0.355	0.043	1.426	1.012	2.009
Age	-0.039	0.000	0.962	0.951	0.973
BMI	0.022	0.050	1.022	1.000	1.044
Dexamethasone initiation (late)	-0.555	0.016	0.574	0.366	0.900
Dexamethasone dose	-0.520	0.290	0.594	0.227	1.558
Dexamethasone duration	0.121	0.608	1.129	0.711	1.793
Route of administration (intravenous vs. other)	-0.880	0.000	0.415	0.292	0.588
O_2_ requirement	-1.523	0.134	0.218	0.030	1.602
Ventilation requirement	-2.653	0.000	0.070	0.044	0.114

## Discussion

The present study was conducted to assess the initial timing of administering corticosteroid therapy and its effects on morbidity, mortality, and hospital stay of severe COVID-19 patients. The results showed a significant difference in outcomes, such as discharge rates and mortality, favoring those who received dexamethasone compared to those who did not. Our findings are consistent with previous studies, which have shown that corticosteroid administration resulted in lower mortality compared to invasive mechanical ventilation or oxygen alone [4]. Individuals suffering severely from COVID-19 experience difficulty in respiration and therefore require ventilatory support. Li et al. (2020) showed that in severe cases, non-invasive ventilation is used if tolerated (invasive ventilation requires skilled intubation by a protected physician) [13]. Corticosteroids, initially controversial, have shown benefits in severe COVID-19 cases, particularly when administered early during the hyperinflammatory phase [14].

Furthermore, the study also showed that the time dexamethasone started had a big impact on patients’ outcomes. In the present study, late initiation of dexamethasone was associated with nearly doubled odds of mortality (Exp(B) = 1.935, p = 0.005). This supports findings from the RECOVERY trial, that in patients who require respiratory support, giving dexamethasone significantly reduced their chances of dying [15]. Similarly, Al Sulaiman et al. (2022) demonstrated through their recent report that critically sick COVID-19 patients who were not yet on mechanical ventilation and were given dexamethasone within one day of their ICU admission were able to avoid the use of mechanical ventilation [16]. However, a study by Sugimoto et al. (2023) showed some opposite findings that those who started their dexamethasone treatment early had more severe health risk factors and a higher rate of death than those who initiated their treatment late (13.3% vs. 7.9%, p < 0.001) [17]. Thus, our study reinforces the importance of timely intervention in managing severe COVID-19 cases. In the present study, the binary logistic regression model revealed that males had a higher likelihood of being discharged home and a lower risk of mortality compared to females. This finding is in contrast with previous studies that suggest gender differences in immune response and disease severity. For instance, a study by Salam et al. (2021) indicated that males might have a higher risk of severe outcomes due to different immune responses and a higher prevalence of comorbidities [18]. Similarly, another study by Raimondi et al. (2021) shows that hospitalized women due to severe COVID-19 are less likely to die from COVID-19 [19].

Moreover, age was a critical factor in predicting outcomes. Older patients had significantly higher mortality rates, with each additional year increasing the odds of mortality. This aligns with the evidence that age is a significant risk factor for severe COVID-19 outcomes. A study by Romero et al. (2021) shows that an increase in age and different comorbidities in patients are risk factors for COVID-19 severe outcomes, such as hospitalization and mortality [20]. Moreover, in previous literature, it is mentioned that older adults are at increased risk for severe illness, likely due to age-related changes in immune function and higher incidence of comorbidities. Likewise, Gray-Miceli et al. (2023) opine that age-related immune changes increase the risk for viral infections such as COVID-19 [21]. Higher BMI was associated with a slightly reduced risk of mortality and increased likelihood of discharge. Previous literature shows mixed results regarding the impact of BMI on COVID-19 outcomes [22-24]. While some research suggests that obesity increases the risk of severe disease and mortality, AlBahrani et al. (2023) show that obesity in COVID-19 patients is linked to lower oxygen saturation (<93%), increased lung infiltrates, higher mechanical ventilation demand, and elevated mortality rates [22]. Moreover, Habis et al. (2023) show that obesity is significantly correlated with poor clinical outcomes in COVID-19. It is also associated with higher mortality and the need for mechanical ventilation necessitating intensive care unit admission [23]. Our findings indicate a nuanced relationship where moderate obesity might not adversely affect outcomes to the extent previously believed. However, a study by Putot et al. (2024) shows that for a cohort hospitalized for COVID-19, low BMI, rather than high BMI, appears as an independent risk factor for death after COVID-19 [24]. However, it is important to note that extreme obesity still poses significant health risks.

The dose of dexamethasone did not significantly predict outcomes, which might suggest that the 6 mg dose typically given in clinical settings is appropriate and effective. This is similar to the RECOVERY study that used 6 mg as the reference dose. However, patients who got more than 6 mg had a worse outcome, so it is very likely that higher doses have the worst impact. Similarly, Horby et al. (2021) show that the RECOVERY trial has some solid evidence that using dexamethasone, at a dose of 6 mg once a day for up to 10 days, can actually lower the mortality rate by 28 days for COVID-19 patients who need respiratory support. So, basically, it shows that this treatment can help save lives [4]. Moreover, the route of dexamethasone administration significantly impacted outcomes. Intravenous administration was associated with higher mortality and lower discharge rates compared to oral or intramuscular routes. This finding is somewhat surprising, as intravenous administration is often considered for more severe cases requiring rapid intervention. However, it might reflect the severity of illness in patients who received intravenous therapy rather than the administration route itself. Wagner et al. (2022) show that systemic corticosteroids probably slightly reduce all‐cause mortality up to 30 days in people hospitalized because of symptomatic COVID‐19 [25]. Oxygen and ventilation requirements were strong predictors of outcomes. Most of the patients needing oxygen had a much higher death rate, but the ones that also needed ventilators had the highest with an odds ratio of 17.620. This shows that the novel findings complement the known ones, which include that the virus affects the respiratory system before invading other organs. A study by Melamed et al. (2022) shows that respiratory failure requiring mechanical ventilation in COVID-19 patients is associated with high mortality [26].

Currently, dexamethasone is included on the WHO's list of essential medicines, making it widely accessible and affordable for the treatment of COVID-19. Guidelines from major health authorities, including the U.K. Chief Medical Officers, the European Medicines Agency, the WHO, and the National Institutes of Health in the United States, have been revised to endorse the use of glucocorticoids for hospitalized patients with COVID-19 who require oxygen, whether or not they are on ventilatory support [4]. Our research indicates that administering dexamethasone early in the treatment process may enhance these benefits, offering further advantages for individuals battling COVID-19.

Limitations

This study is not without limitations. The observational nature of the study limits the ability to establish causality. Potential confounders, such as underlying comorbidities and variations in healthcare delivery, might influence the results. Additionally, our study is based on a single center, which might limit the generalizability of the findings to other settings or populations.

Future directions

Our study highlights the importance of an early start with dexamethasone as well as the need for individual treatment techniques in the fight against COVID-19. Gender and BMI and their complex impact on different cases are areas of future research to allow the implementation of therapeutic interventions tailored to the diverse patient mix and ultimately improve patients' prognosis.

## Conclusions

Our study sheds light on the important factors that affect outcomes in severe COVID-19 patients. It shows that starting dexamethasone treatment early is crucial, along with using the right administration routes. In addition, we found that age, gender, and BMI also play a big role. These findings add to the growing knowledge about how to manage COVID-19 and highlight the need for personalized treatment strategies.
